# Integration of metabolomics data into metabolic networks

**DOI:** 10.3389/fpls.2015.00049

**Published:** 2015-02-17

**Authors:** Nadine Töpfer, Sabrina Kleessen, Zoran Nikoloski

**Affiliations:** ^1^Systems Biology and Mathematical Modeling Group, Department Willmitzer, Max-Planck Institute of Molecular Plant PhysiologyPotsdam, Germany; ^2^Department of Plant Sciences, Weizmann Institute of ScienceRehovot, Israel; ^3^Targenomix GmbHPotsdam, Germany

**Keywords:** constraint-based modeling, metabolomics, data integration, model reconstruction, flux prediction

## Abstract

Metabolite levels together with their corresponding metabolic fluxes are integrative outcomes of biochemical transformations and regulatory processes and they can be used to characterize the response of biological systems to genetic and/or environmental changes. However, while changes in transcript or to some extent protein levels can usually be traced back to one or several responsible genes, changes in fluxes and particularly changes in metabolite levels do not follow such rationale and are often the outcome of complex interactions of several components. The increasing quality and coverage of metabolomics technologies have fostered the development of computational approaches for integrating metabolic read-outs with large-scale models to predict the physiological state of a system. Constraint-based approaches, relying on the stoichiometry of the considered reactions, provide a modeling framework amenable to analyses of large-scale systems and to the integration of high-throughput data. Here we review the existing approaches that integrate metabolomics data in variants of constrained-based approaches to refine model reconstructions, to constrain flux predictions in metabolic models, and to relate network structural properties to metabolite levels. Finally, we discuss the challenges and perspectives in the developments of constraint-based modeling approaches driven by metabolomics data.

## Introduction

The metabolome comprises the complete set of metabolites, the non-genetically encoded substrates, intermediates, and products of metabolic pathways, associated to a cell (Nielsen and Jewett, 2007). While RNA and proteins are encoded in the DNA, the variety of metabolites with their particular chemical properties is immensely large and cannot be directly inferred from the genome (Lenz and Wilson, 2007). Therefore, metabolites can be regarded as the bridging component between the genotype and the phenotype (Fiehn, 2002).

Recent years have witnessed the development and application of metabolomics technologies that facilitate large-scale identification and quantification of metabolites. These technologies complement the well-established methodology used in genomics, transcriptomics, and proteomics studies which are marked by a large coverage of the respective cellular components (Romero et al., 2006). The integration of data generated from these high-throughput platforms holds the promise to ultimately help determine the gene-function relationship (Nobeli and Thornton, 2006)—the long-standing goal of modern biology.

Metabolome analysis aims at identifying and quantifying the entire collection of metabolites in a biological system (Oliver et al., 1998). As the spectrum of metabolites is extremely wide in concentration and physico-chemical properties, no single methodology can facilitate the simultaneous measurement of the entire metabolome (Nobeli and Thornton, 2006). Therefore, the term metabolomics refers to a collection of technologies which cover different parts of the metabolome (Redestig et al., 2011). Usually, metabolomics studies report relative quantifications of metabolites, determined by the fold-change (unitless number) of the peak size between two samples. In comparison, absolute metabolite quantifications require calibration curves of standards for each metabolite, and result in levels given in moles per weight of tissue, *e.g.*, mol per gram (g) fresh weight (FW). While relative changes in metabolite levels provide sufficient information for many applications, there is an increasing focus on the determination of absolute metabolite levels. For a comprehensive review about the spectrum of metabolomics approaches we refer to Dettmer et al. (2007) and Kueger et al. (2012).

Since metabolites are embedded in an intricate network structure, the metabolome can be regarded not only as a connecting component between the genotype and the phenotype, but also as a cellular level in its own right. Large-scale studies of metabolism have shifted focus from the analysis of the structure of the underlying network, driven by progress in complex network research (Jeong et al., 2000; Parter et al., 2007), to understanding the relation of metabolic processes to other cellular levels affecting them (*e.g.*, transcriptional and translational regulation, Chandrasekaran and Price, 2013; Scott et al., 2014, as well as protein abundances and turnover, O'Brien et al., 2013). Formal large-scale analysis of metabolism, even under simplifying assumptions about the laws governing the transformation of molecules, is particularly challenging due to the nonlinearities in the underlying relationships. As metabolomics data are read-outs from complex interaction networks, their analysis in a network context can reveal the underlying network structure and regulation. Two main approaches are usually applied to model metabolic networks—kinetic approaches and stoichiometry-based methods.

Classical kinetic modeling approaches describe the rate of change in the concentration of the considered metabolites based on the enzyme kinetics (*e.g.*, mass action or its derivative, Michaelis-Menten) with the corresponding parameters (*e.g.*, rate constants or phenomenological constants, such as maximum reaction velocity *V*_*max*_ and dissociation (Michaelis-Menten) constant *K*_*m*_). Therefore, the solution of the resulting system of ordinary differential equations, *dX*/*dt* = *N* · *v*(*X*, *p*), with *X* representing the concentration of metabolites, *N* representing the stoichiometric matrix, *v* the vector of metabolic fluxes (*i.e.*, rates, velocities), and *p* standing for the various parameters, yields the concentration-time trajectories of the metabolites. These approaches have successfully been applied to study small and moderate-sized metabolic networks (for general reviews see Resat et al., 2009; Machado et al., 2011).

However, the advances in high-throughput technologies during the last two decades paved the way for large-scale metabolic network reconstructions which aim at providing an integrated view of an organism's metabolism. These models not only represent the stoichiometry of several hundred to several thousand metabolic reactions in the stoichiometric matrix but they also contain a mathematical representation of the gene-reaction relationship. For example, this annotation makes it possible to *in silico* study the phenotype of gene knockouts or to integrate transcriptomics data (for reviews see Blazier and Papin, 2012; Lewis et al., 2012). Moreover, a comprehensive overview of the generation of genome-scale models can be found in Thiele and Palsson (2010) and Henry et al. (2010). As a kinetic description of the behavior of these large networks is hampered by uncertainties in both, the underlying kinetics and the respective parameters, a large collection of stoichiometry-based (often also referred to as constraint-based) approaches have been developed in parallel with genome-scale models. These approaches are derived from the classic Flux Balance Analysis (FBA) formulation (Varma and Palsson, 1994a; Orth et al., 2010, and also see Table 1) and have in common that they solely rely on the stoichiometry of the network, given chemico-physical constraints, and an optimization goal under which the organism is considered to operate. For example, for microorganisms this optimization goal, or the so called objective function, is usually the maximization of growth (Feist and Palsson, 2010). For other systems, such as blood cells or plants, the minimization of fluxes or photon usage was introduced as an alternative principle (Holzhütter, 2004; De Oliveira Dal'Molin et al., 2010). Moreover these FBA-based methods assume that changes on the metabolic level happen so fast that the system under consideration can be considered to be in a steady-state (Varma and Palsson, 1994b):

$$\frac{dX}{dt}=N\cdot v(X,p)=0.$$

**Table 1:** **Mathematical formalisms in computational biology used throughout this review**.

**Formalism**	**Mathematical formulation**
**Flux balance analysis—FBA (Varma and Palsson, 1994c)**	max *c*^*T*^ · *v*
This computational approach predicts steady-state flux distributions that are thermodynamically feasible and mass-balanced. The underlying assumption of the method is that the organism under consideration operates under a certain optimality goal, *e.g.*, the maximization of growth for microorganisms. The optimization problem formulation relies solely on the stoichiometry of the participating reactions (represented in the stoichiometric matrix *S*), lower and upper boundaries (*v*_*min*_ and *v*_*max*_) for the respective fluxes and an optimization goal (captured in the vector *c*) as input. No knowledge of initial metabolite concentrations or kinetic parameters is required.	*s*.*t*.$\frac{dx}{dt}$ = *S* · *v* = 0
	*v*_*min*_ ≤ *v* ≤ *v*_*max*_
**Minimization of metabolic adjustment—MOMA (Segrè et al., 2002)**	*min* (*v* − *w*)^2^
This FBA-based method was developed to identify a feasible flux distribution of a genetically perturbed system which is closest to the wild-type flux distribution. The rationale for this approach is the assumption that the metabolic network of the organism under consideration adjusts to the perturbation with a minimal rewiring of the flux profile. The set *A* contains all reactions which are switched off in the perturbed organism. Due to the introduction of the Euclidean norm the resulting optimization problem is quadratic.	*s*.*t*.$\frac{dx}{dt}$ = *S* · *v* = 0
	*v*_*min*_ ≤ *v* ≤ *v*_*max*_
	*v*_*k*_ = 0, *k* ∈ *A*
**Regulatory on/off minimization—ROOM (Shlomi et al., 2005)**	min $\sum_{i\;=\;1}^{m}y_{i}$
Similar to MOMA, this method aims at predicting steady-state flux distributions in a perturbed system which are closest to the wild-type flux distribution. However, ROOM relies on the minimization of the number of significant changes of fluxes (hence on/off) with respect to the wild type. The thresholds determining significant flux changes are given by *w*^*U*^ and *w*^*L*^ for upper and lower bounds, respectively. They are defined by relative (δ) and absolute (ε) ranges of tolerance from the wild type fluxes. For each flux *v*_*i*_, 1 ≤ *i* ≤ *N*, *y*_*i*_ = 1 for a significant flux change in flux *v*_*i*_ and *y*_*i*_ = 0 otherwise. The introduced binary variables (*y*_*i*_) render the problem a mixed-integer linear problem (see below).	*s*.*t*.$\frac{dx}{dt}$ = *S* · *v* = 0
	*v*_*min*_ ≤ *v* ≤ *v*_*max*_
	*v*_*k*_ = 0, *k* ∈ *A*
	∀_*i*_1 ≤ *i* ≤ *m*
	*v*_*i*_ − *y*_*i*_ (*v*_*max,i*_ − *w*^*U*^_*i*_) ≤ *w*^*U*^_*i*_
	*v*_*i*_ − *y*_*i*_ (*v*_*min,i*_ − *w*^*L*^_*i*_) ≥ *w*^*L*^_*i*_
	*y*_*i*_ ∈ {0, 1}
	*w*^*U*^_*i*_ = *w*_*i*_ + δ|*w*_*i*_| + ε
	*w*^*L*^_*i*_ = *w*_*i*_ − δ|*w*_*i*_| − ε
**Dual formulation**	**Primal:**
The duality theorem states that for every Primal optimization problem, there exists a Dual problem. In general, the solution to the Dual problem provides a lower bound to the solution of the Primal (minimization) problem. For convex optimization problems the value of an optimal solution of the Primal problem is given by the value of an optimal solution of the Dual problem (Boyd and Vandenberghe, 2004). Here, *x* and *y* is the vector of the Primal/Dual variable, respectively.	max *c* · *x*
	*s*.*t*. *S* · *x* = *b*
	*x* ≥ 0
	**Dual:**
	min *b* · *y*
	*s*.*t*. *y* · *S* = *c*
	*x* ≥ 0
**Convex vs. non-convex optimization**	
For a convex optimization problem, if it is feasible, there can only be one optimal solution, which is globally optimal. Linear programming problems are always convex problems. Non-convex optimization may have multiple local optima. Hence, convex optimization problems can be much faster and more efficiently solved than non-convex optimization problems.	
**Linear programming (LP)**	min *c*^*T*^ · *x*
Optimization problem in which the objective and all constraints are linear. If the vector of variables *x* includes entries which are only allowed to be integers the problem changes into a Mixed-integer linear programming (MILP) problem.	*s*.*t*. *Ax* ≤ *b*
	*x*_*min*_ ≤ *x* ≤ *x*_*max*_
**Quadratic programming (QP)**	min $\frac{1}{2}$*x*^*T*^*Q* · *x* + *c*^*T*^ · *x*
Optimization problem in which the objective is a quadratic function and all constraints are linear.	*s*.*t*. *Ax* ≤ *b*
	*x*_*min*_ ≤ *x* ≤ *x*_*max*_
**Nonlinear programming (NLP)**	min *f*(*x*)
Optimization problem in which the objective and/or constraints are nonlinear. If the vector of variables *x* includes entries which are only allowed to be integers the problem changes into a Mixed-integer nonlinear programming (MINLP) problem.	*s*.*t*. *g*(*x*) ≤ *b*
	*x*_*min*_ ≤ *x* ≤ *x*_*max*_

The steady-state assumption allows solving the system of linear equations, *N* · *v* = 0, for the metabolic fluxes. Nevertheless, despite the resulting decoupling of fluxes and metabolite concentrations in classical stoichiometry-based approaches, in recent years elaborate methods have been developed to facilitate the integration of not only metabolomics data but also the plethora of high-throughput data from other levels of the cellular organization.

**Figure 1:**
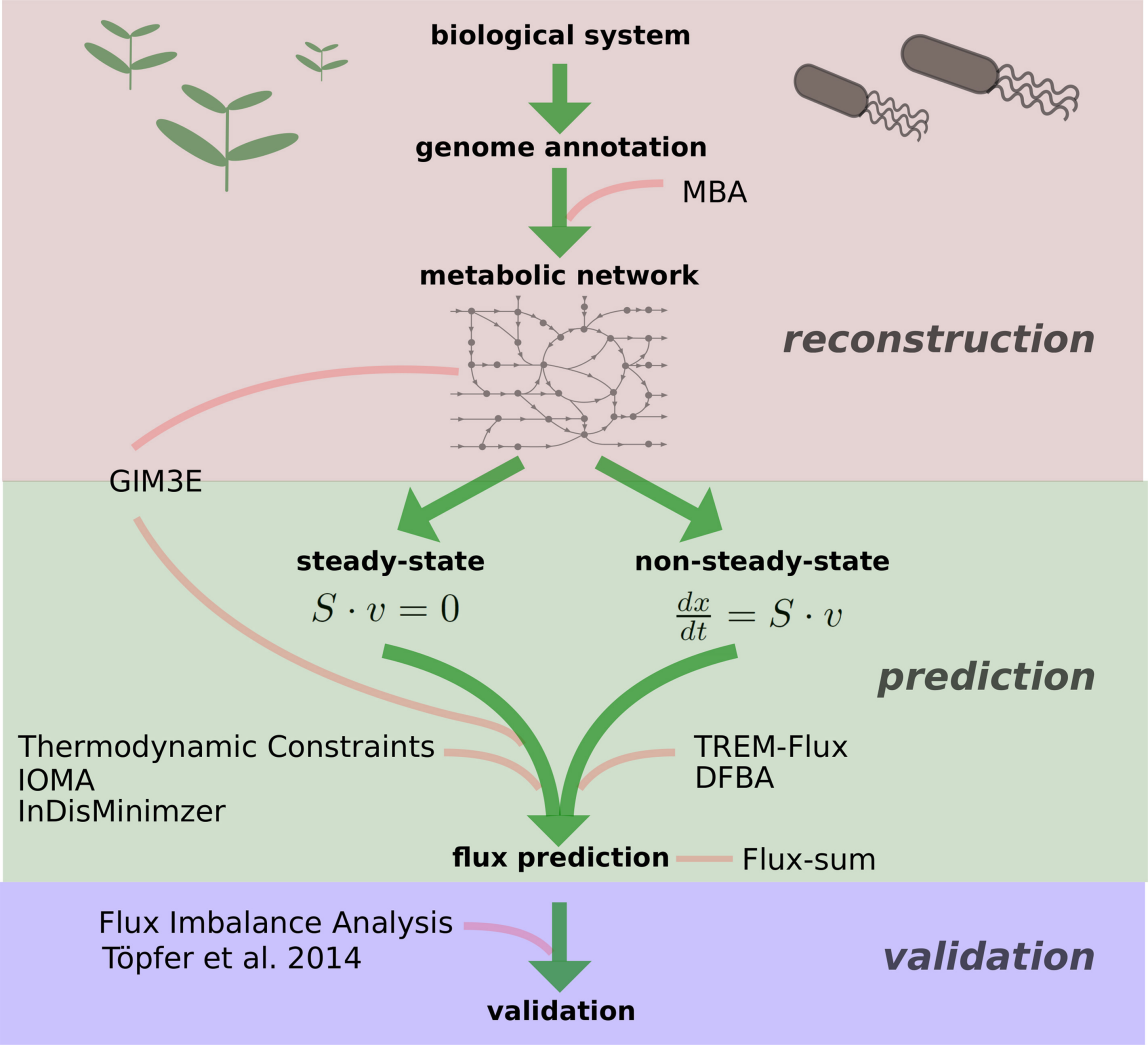
**Schematic overview of the described approaches.** Depicted are the different levels and methods at which constraint-based approaches integrate metabolite data—starting from the model reconstruction to the validation of experimental observations. MBA, Model Building Algorithm (Jerby et al., 2010); GIM3E, Gene inactivation Moderated by Metabolism, Metabolomics, and Expression (Schmidt et al., 2013); IOMA, Integrative Omics-Metabolic Analysis (Yizhak et al., 2010); InDisMinimzier, Integrative Discrepancy Minimizer, (Recht et al., 2014); TREM-Flux, Time-Resolved Expression and Metabolite-based prediction of flux values; DFBA, Dynamic Flux Balance Analysis (Mahadevan et al., 2002).

In this comprehensive systematic review, we present constraint-based approaches that make use of metabolite data to refine model reconstructions, to constrain flux predictions in metabolic network models, and to relate network structural properties to metabolite levels (see Table 2 and Figure 1). We particularly focus on plant-specific studies that make use of the covered approaches. Finally, we discuss current limitations and challenges in data generation, method development, and their coupling in applications.

**Table 2:** **Overview of methods that integrate metabolite levels at various levels**.

**Method**	**Modeling task**	**Contribution**	**References**	**Organism**	**First application**	**Plant application**
Model building algorithm (MBA)	Reconstruction	Reconstruction of tissue-specific models based on metabolite detection in different tissues	Jerby et al., 2010	*H. sapiens*	Reconstruction of a tissue-specific network from a generic human metabolism model	Mintz-Oron et al., 2012 Reconstruction of a tissue-specific network of *A. thaliana*
Gene inactivation moderated by metabolism, metabolomics, and expression (GIM3E)	Reconstruction/flux prediction	Adding turnover metabolites and sink reactions to the generic model	Schmidt et al., 2013	*S. typhimurium*	Investigation of metabolite turnover and integration of transcriptomics data	
Integrative omics-metabolic analysis (IOMA)	Flux prediction/kinetics	Michaelis-Menten-like kinetic	Yizhak et al., 2010	*E. coli*	Analysis of genetic perturbations in *E. coli*	
Dynamic flux balance analysis (DFBA)	Flux prediction	Parameterization of dynamic equations	Mahadevan et al., 2002	*E. coli*	Dynamics of diauxic growth of *E. coli* on acetate and glucose	Dynamics of photosynthetic metabolism in C3 plants by M-DFBA (Luo et al., 2009)
Thermodynamically realizable flux minimization	Thermodynamics	Determination of thermodynamically feasible concentration ranges	Hoppe et al., 2007	*H. sapiens*	Analysis of a small-scale network of red blood cells	
Integrative discrepancy minimizer (InDisMinimizer)	Flux prediction	Deriving of flux rates for the accumulation of metabolite groups	Recht et al., 2014	*H. pluvialis*		Study of stress-induced carbon re-partitioning in *H. pluvialis*
Time-resolved expression and metabolite-based prediction of flux values (TREM-Flux)	Flux prediction	Replacement of steady-state assumption to the requirement that changes in flux distributions coincide with differences in metabolite levels	Kleessen et al., 2015	*C. reinhardtii*		Metabolic response of *C. reinhardtii* to rapamycin treatment
Flux imbalance analysis	Validation	Sensitivity of metabolic optima to violations of the steady-state constraints	Reznik et al., 2013	*S. cerevisae*	Analysis of nutrient limiting conditions in *S. cerevisae*	
Linking metabolite levels to differential metabolic pathways	Validation	Linking of flux predictions from transcriptomics data to metabolite levels	Töpfer et al., 2014	*A. thaliana*		Analysis of the response of *A. thaliana* to environmental stress conditions
Flux-sum	Flux prediction	Descriptor of a turnover rate of a metabolite used to investigate the metabolic state	Chung and Lee, 2009	*E. coli*	Investigation of the type of metabolite essentiality in *E. coli*	Analysis of two different nitrogen conditions in maize leaf

## Metabolite data to reconstruct tissue-specific networks

### Model building algorithm

The Model Building Algorithm (MBA) makes use of metabolites that were detected in a given organ or tissue (Jerby et al., 2010). In its first application, a liver metabolomics data set was used for the reconstruction of tissue-specific networks from a generic human metabolism model. The metabolomics data are employed in combination with other tissue-specific data, such as: literature-based knowledge, transcriptomics, proteomics, and phenotypic data, to define two sets of reactions—high-probability (*C*_*H*_) and moderate probability reactions (*C*_*M*_). High-probability reactions are those that are part of human-curated tissue-specific pathways. A reaction is considered to be a member of the group of moderate probability reactions only if it was necessary for the inclusion of a liver metabolite that appeared in the metabolomics data or if it was supported by at least two of the used data sources.

The subsequent optimization procedure employs a greedy heuristic search algorithm to arrive at the most parsimonious tissue-specific consistent model that guarantees the inclusion of all the tissue-specific reactions in *C*_*H*_, a maximum number of reactions from *C*_*M*_, and a set of additional reactions from the generic model that are necessary for gap filling. Cross validation was used for model selection by leaving out core reactions as well as data sets to predict hepatic flux measurements. Finally, the derived liver model was validated by simulating various known hepatic metabolic pathways. Predictions of metabolic biomarkers demonstrated that the resulting model performed better than the underlying generic model.

#### In planta—application of the MBA for extracting tissues-specific Models of Arabidopsis from a generic model

MBA was used to extract 10 tissue-specific metabolic networks (*i.e*., culture light, culture dark, silique, flower bud, open flower, root 10 days, root 23 days, juvenile leave, cotyledon, and seed) from a generic model of *Arabidopsis thaliana* (Mintz-Oron et al., 2012). The authors slightly adapted the method to fit plant-specific modeling needs. First, they allow not only for the addition of generic reactions to the set of core reactions, but also for the relaxation of irreversibility of existing core reactions, if this increases the set of activated core reactions. In addition, reactions from the generic model are prioritized based on their organism of origin, *i.e*., reactions from *Arabidopsis* or other closely related organisms are more likely to be included than reactions from distant organisms. Finally, a constraint is introduced that enforces the production of all biomass compounds under minimal media.

### Gene inactivation moderated by metabolism, metabolomics, and expression (GIM3E)

Another approach for context-specific network extraction is Gene Inactivation Moderated by Metabolism, Metabolomics, and Expression (GIM3E) (Schmidt et al., 2013). GIM3E is an extension to GIMME (Becker and Palsson, 2008), a network extraction approach that integrates transcriptomics data to derive penalty coefficients for the considered reactions and to subsequently compute condition-specific models of smallest penalty score. GIM3E integrates metabolomics data by adding turnover metabolites and a respective sink reaction to the generic model. Therefore, a flux through a respective metabolic reaction can be obtained, and experimentally detected metabolites can be integrated by enforcing a minimum flux for the turnover of the respective metabolite. By doing so, the authors are able to enforce fluxes though reactions without violating the steady-state assumption. This approach relies on a cellular objective, *e.g.*, the maximization of biomass, whose optimal value is used as an additional constraint for the enforcement of the turnover fluxes. More specific, in their study the authors required the network to produce 99% of the optimum value of biomass while enforcing a small positive flux value through turnover sink reactions of the corresponding experimentally detected metabolites.

The algorithm was developed to study the metabolism of the bacterium *Salmonella typhimurium*, particularly to allow for the investigation of metabolite turnover in two scenarios, namely, rich and virulence media. In a similar fashion to MBA, the approach has the advantage that it can be employed to gain insights about metabolite turnover rates in the system under investigation. A potential drawback of the approach lies in the nature of the problem formulation. The method relies on converting all reversible reactions to reaction pairs of forward and backward reactions; the applied constraints, *i.e*., the decision whether the forward or backwards reaction of a reaction pair is activated, result in a mixed integer linear program (MILP) which becomes time-demanding for very large networks.

## Bridging the gap between kinetic modeling and stoichiometry-based approaches

### Including kinetic information into FBA

#### Integrating quantitative proteomics and metabolomics with a genome-scale metabolic network model (IOMA)

Yizhak et al. (2010) presented an approach that seeks a steady-state flux distribution through a metabolic network that is most consistent with flux estimations which are derived from the integration of quantitative metabolomics and proteomics data by assuming an underlying Michaelis-Menten-like kinetic. The chosen functional form also enables the integration of proteomics data, which represent relative protein levels compared to some reference state as well as absolute metabolite data to arrive at flux values. These estimates are integrated in the overall flux prediction by solving a quadratic program (QP), which finds a feasible flux distribution that is as consistent as possible with the rates derived from the data.

The approach was validated via two comparative analyses: First, the predictive performance of the method was compared to that of Minimization of Metabolic Adjustment (MOMA) a method commonly used to identify a feasible flux distribution of a perturbed system which is closest to the wild-type flux distribution (Segrè et al., 2002) (see Table 1 for a description of the approach). This comparison is based on predicted flux distributions upon gene knock out simulations which were generated using a kinetic model of red blood cells and randomly generated proteomics data. Second, the performance of the approach was compared to those of MOMA and FBA in studies of genetic perturbations in *E. coli* for which proteomics, metabolomics, and flux measurements were available.

The advantage of the approach lies in the program formulation which seeks to minimize errors introduced from noisy data or from the simplified Michaelis-Menten-like enzyme kinetic. The assumed kinetic law may pose disadvantages: While it may be the case that not all enzymes follow the assumed kinetics, besides metabolite abundances the approach also requires that relative protein abundances, metabolite dissociation constants and *V*_*max*_ values are available. While the first two are specific for the respective experiment, the latter two might be obtained from the literature. Although new types of experimental data are becoming increasingly available (Tummler et al., 2013), the applicability of this approach remains limited to well-studied organisms for which these kinetic information are collected.

### Dynamic optimization approaches

#### Dynamic flux balance analysis allows prediction of dynamics with only limited knowledge of kinetic parameters

As indicated above, the dynamics of metabolic networks can be investigated by kinetic modeling. To this end, the parameters of specific enzyme kinetics have to be determined by measurements of enzyme activities and data fitting to experimentally obtained metabolite concentrations. The requirements of this large amount of data limit the application of kinetic modeling methods only to well-studied systems of moderate scale and complexity (Rios-Estepa and Lange, 2007; Nägele et al., 2010; Rohwer, 2012). In contrast, Mahadevan et al. presented Dynamic Flux Balance Analysis (DFBA) as an alternative to predict time-resolved metabolite levels and flux distributions with only a limited knowledge of enzyme kinetics (Mahadevan et al., 2002).

DFBA overcomes the main drawback of the classical FBA which precludes the analysis of the dynamic behavior of a network—the steady-state assumption. Within DFBA, (time-resolved) measurements of metabolite levels can be directly integrated to obtain more accurate flux predictions. Two general DFBA formulations were introduced—static and dynamic (Mahadevan et al., 2002). The static optimization approach (SOA), which results in a LP, involves first dividing the time period of interest into uniform time intervals and then solving the instantaneous optimization problem at the beginning of each time interval, followed by integration to compute metabolite concentrations over time. The SOA considers a steady-state and therefore only allows changes in external metabolite concentrations. On the other hand, the general dynamic optimization approach (DOA) involves optimization over the entire time period by parameterizing the dynamic equations with the help of orthogonal collocation on finite elements (OCFE) (Cuthrell and Biegler, 1987). An illustrative tutorial of OCFE can be found in Kleessen and Nikoloski (2012). The DOA, which allows, in addition, to analyze internal metabolite concentrations, usually results in a non-linear program (NLP) due to nonlinear constraints.

Mahadevan and coworkers used both alternatives of DFBA to predict the dynamics of diauxic growth of *E. coli* on acetate and glucose. While classical FBA incorrectly predicted the reutilization of acetate (Varma and Palsson, 1994c; Mahadevan et al., 2002) DFBA provides a significant improvement due to the ability to characterize different phases of batch growth which qualitatively match experimental results.

Moreover, the DOA variant of DFBA has been combined with MOMA (Segrè et al., 2002), resulting in the so-called M-DFBA approach. In their classical formulations, both, FBA and DFBA, ignore the possibility that under perturbed conditions metabolic network may not be regulated toward the generally considered optimal objective. To this end, MOMA was designed based on the hypothesis that fluxes in perturbed metabolic networks undergo a minimal redistribution compared to those of the unperturbed network. Similarly, in M-DFBA minimal fluctuation of the dynamic profile of metabolite levels over time is considered as objective to represent the behavior in perturbed systems (Luo et al., 2006). Luo and coworkers applied M-DFBA to explore the dynamic adjustment of the mammalian myocardial energy metabolism under normal and ischemic conditions. The predictions from M-DFBA were able to represent the dynamic regulation of utilization of metabolic substrates for energy production more accurately than the classical DFBA assuming maximal ATP production as an objective under ischemic conditions. This supported the assumption that under perturbed conditions a system does not follow the assumed objective but instead reaches a suboptimal level of energy production.

#### In planta—Dynamic flux balance analysis reveals time-resolved behavior of plant systems

Alongside the applications of different variants and slight modifications of DFBA in non-photosynthetic organisms (Lee et al., 2008; Krauss et al., 2012), DFBA-based methods have been widely employed for predicting the dynamics of plant systems. In its first application, Luo et al. (2009) studied the photosynthetic metabolism in C3 plants and posited hypotheses about its robustness under different CO_2_ and water conditions by using the M-DFBA approach.

In addition, a suite of DFBA-based approaches using DOA has been proposed to analyze the dynamics of (internally perturbed) metabolic networks and for quantifying their robustness with only a limited knowledge of kinetic parameters (Kleessen and Nikoloski, 2012). This suite consists of variants of DFBA, M-DFBA as well as a new proposed coupling of the principle of Regulatory on/off minimization (ROOM) (Shlomi et al., 2005) (see Table 1 for a description of the approach) with DFBA (R-DFBA). As a contending alternative for MOMA, ROOM relies on significant flux changes, extended in R-DFBA to minimize of the total number of significant changes of metabolite concentrations over time (also fluxes in some variants). In total 10 different DFBA-based approaches were analyzed of which seven were newly proposed. By conducting a comparative analysis of the different variants with a kinetic model of the Calvin-Benson cycle and a model of plant carbohydrate metabolism, it was shown that DFBA-based methods can accurately predict the changes in metabolic states. Therefore, DFBA and its extensions are suitable for positing model-based hypotheses for the dynamics of metabolic pathways when only little enzymatic details are known (Kleessen and Nikoloski, 2012).

Furthermore, a DFBA-based approach was developed to investigate different model variants of the mitochondrial electron transport chain (ETC) in *A. thaliana* during dark-induced senescence in order to elucidate alternative substrates for this metabolic pathway (Kleessen et al., 2012). The findings demonstrated that the coupling of the proposed computational approach with measured time-resolved metabolomics data results in model-based confirmations of the given hypotheses. This approach can also help to find modified pathways at different levels of plant adaptation to various conditions.

In contrast, Grafahrend-Belau et al. (2013) used the static variant of DFBA in combination with a multiscale modeling approach to achieve the spatiotemporal resolution of source-sink interactions in barley (*Hordeum vulgare*) by integrating static organ-specific models with a whole-plant dynamic model. However, the static variant of DFBA is restricted to integrate only metabolite levels of import and export reactions from the analyzed system while in the dynamic variant also internal metabolite levels can be included as well as kinetic expressions. To this end, DOA variants of DFBA can predict flux and metabolite levels even beyond the measured time points.

Nevertheless, due to the computationally intense formulation in terms of orthogonal collocation on finite elements, requiring a large number of variables, the application of DFBA-based is currently restricted to relatively small metabolic networks. The underlying mathematical problem of DFBA results in a combinatorial explosion in the number of unknown variables as the network size increases.

## Metabolite data to infer thermodynamic realizability

### Including metabolite concentrations into flux balance analysis

Metabolite concentrations in metabolic networks are intrinsically tied to the thermodynamic potential of Gibbs Energy Δ*G*, given by:

$$\Delta G=\Delta {\text{G}}^{0}+RT\sum \ln\;P-RT\sum \ln\;S,$$

where Δ*G*^0^ is the standard Gibbs Energy, *R* is the universal gas constant, *T* the temperature, and *P* and *S* are the product and the substrate concentrations of the reaction, respectively. A negative Gibbs Energy indicates that the respective reaction proceeds in the forward reaction, whereas a positive Gibbs Energy indicates that the reaction is proceeding backwards. Several approaches make use of these information to either integrate metabolite levels or estimates of concentration ranges, or to infer these information form the models predictions (Holzhütter, 2004; Kümmel et al., 2006; Henry et al., 2007).

### Thermodynamic realizability as a constraint on flux distributions in metabolic networks

Here, we briefly describe the method by Hoppe et al. (2007) which makes use of metabolite concentration ranges in the abovementioned manner to predict more reliable flux distributions than a generic model. The objective function of this approach is a combination of flux minimization and minimizing penalties that arise from violating thermodynamically feasible concentration ranges. The approach results in the solution of a mixed integer linear optimization problem with a quadratic objective function. The algorithm computes an optimal flux distribution and a metabolite profile which is thermodynamically feasible and assures minimal deviations of the respective metabolite levels from their expected values, the so-called thermodynamically realizable flux-minimized solution.

The authors used the approach to study a small-scale network of red blood cells and a large-scale network of *E. coli* and demonstrate that increasing network complexity results in increased sensitivity of the predicted fluxes to variations of standard Gibbs Energies and metabolite concentration ranges. The lack of any additional assumption during the introduction of simple thermodynamic rules liberates the approach from potential errors that might arise from speculations about underlying kinetics. However, like DFBA, the approach depends on the availability of absolute metabolite concentrations or on plausible absolute concentration ranges which may not be readily available (Van der Greef et al., 2003).

### Balancing the maximization of enzyme efficiency and the minimization of total metabolite load—a metabolic tug of war

Tepper et al. (2013) developed a method to compute steady-state metabolite concentrations and flux values in microorganisms on a genome-scale. Their approach, termed “metabolic tug-of-war (mTOW)” suggests an underlying balance between maximizing enzyme efficiency on the one hand and minimizing total metabolite load on the other. The rationale for this assumption weights factors that favor a small metabolite pool size, *e.g*., due to limited capacities of the cell, vs. the need to maintain an adequate thermodynamic driving force, *i.e.*, concentrations far from chemical equilibrium, in order to direct metabolic fluxes. In other words the computational formulation captures the trade-off between minimizing the total concentration of intermediate metabolites and maintaining adequate forward driving force for all reactions based on the laws of thermodynamics. The computational procedure involves estimating Gibbs Energies via Component Contribution Method (CCM) (Noor et al., 2013) followed by a non-convex optimization (for an explanation see Table 1), searching for a flux distribution and metabolite concentrations that minimizes both the metabolite and enzyme levels.

The authors applied their approach in a test study to a set metabolite data from *E. coli* and *Clostridium acetobutylicum* under several growth conditions. They showed that mTOW can explain up to 55% of the observed variation in measured metabolite concentrations in both organisms and therefore, presented the first study that is able to predict high-throughput metabolite concentration data in microorganisms across several conditions. A drawback of the approach is its non-convex nature of the problem formulation. The optimization problem might have multiple optima—local and a global one, whose exact solution is computationally intractable for large-scale networks.

## Metabolite data to analyze flux re-routing

### *In planta*—integrative *discrepancy minimization* reveals metabolic constraints for carbon partitioning under high-light and nitrogen-starvation in the green algae *hematococcus pluvialis (InDisMinimizer)*

A recent method enables constraining condition-specific solution spaces by integrating information about the environment with measured metabolite, enzymology, and physiology data (Recht et al., 2014). In this approach, the experimental data are integrated by deriving flux rates and subsequently minimizing the discrepancy between the experimental data and predictions from the model.

The approach is formulated as a QP, and is applied to study stress-induced carbon re-partitioning in the green algae *H. pluvialis*. Measurements of groups of metabolites, *e.g*., chlorophylls, proteins, total fatty acid content, and total carbohydrate content in combination with dry weight and cell number measurements are used to derive flux rates for the accumulation of these metabolite groups. By introducing sink reactions for these groups of compounds major flux routes in the model can efficiently be constrained. Based on the constrained model, two hypotheses about carbon partitioning under the respective stress condition were tested *in silico* by performing flux variability analyses for different biological scenarios while enforcing a best fit between model and data.

The approach was used to test whether starch can be degraded to supply carbon skeletons as precursors for starvation-induced fatty acid synthesis and to test whether an increased activity of the TCA cycle, observed under the stress condition, can support a high synthesis rate of fatty acids. The findings showed strong model-driven support for the proposed mechanisms and provide the basis for further experimental testing strategies. Similar to the approach presented by Yizhak et al. (2010), the method is free from a biologically motivated objective function (which is difficult to define for organisms that experience challenging conditions) and it accounts for noisy data by minimizing discrepancies between observation and model prediction. A disadvantage of the approach is that the flux rates are derived from time points that span up to 24 h and therefore, only represent average accumulation rates.

## Direct integration of metabolite data to constraint flux predictions

### *In planta*—time-resolved expression and metabolite-based prediction of flux values (*TREM-flux*) specifies chlamydomonas' metabolic response to rapamycin treatment

Since the application of DFBA-based methods to genome-scale models is currently hampered by the model size and the lack of optimization platforms which scale well, a constraint-based method to couple time-resolved transcriptomics and metabolomics data, termed Time-Resolved Expression and Metabolite-based prediction of Flux values has been proposed (Kleessen et al., 2015).

In this approach, the steady-state assumption of the general FBA approach is replaced with the requirement that the changes in flux distribution must coincide with the difference of the measured metabolite levels between two consecutive time points, while matching global physiological parameters. Although post-translational protein modifications may affect the cellular state, in this approach, the more easily accessible and comprehensive transcriptomics data are used to further constrain the time-resolved flux predictions by applying a dynamic variant of the E-Flux method.

In a genome-scale model reconstruction of *Chlamydomonas reinhardtii* TREM-Flux was used to predict the metabolic response to rapamycin treatment. The obtained flux distributions over time showed differences in the metabolic responses under varying growth conditions between control and treatment, in line with the findings from closely related organisms. The study shows that the integration of time-resolved unlabeled metabolomics data in addition to transcriptomics data can specify metabolic pathways involved in the system's response to a treatment.

## Stoichiometry-based analysis provides hints on behavior of metabolite levels

### Flux imbalance analysis

Flux Imbalance Analysis explores the sensitivity of metabolic optima to violations of the steady-state constraints (Reznik et al., 2013). The method does not directly integrate metabolomics data but demonstrates that the underlying mathematical framework can be used to elucidate biologically significant information on the processes that control intracellular metabolite levels. Reznik et al. used the dual formulation (for an explanation see Table 1) of a classical FBA problem to compute sensitivities of the objective value to flux imbalances, *e.g*., deviations from the steady-state assumption. The so-called shadow price of a given metabolite in the dual problem captures the influence of the metabolite's accumulation or depletion on the maximum value of the objective. Thereby, a negative shadow price implies that the corresponding metabolite is growth limiting.

By using data from *Saccharomyces cerevisae* under different nutrient limiting conditions, the authors showed that the determined shadow prices are negatively associated with the growth limitation of the respective measured intracellular metabolites. Moreover, based on these findings, the authors argued that growth-limiting metabolites cannot exhibit large fluctuations in an uncontrolled manner. Using time-resolved metabolomics data from the metabolic response of *E. coli* to carbon and nitrogen perturbations, they further demonstrated that metabolites associated with a negative shadow price indeed show lower temporal variation in comparison to metabolites with zero shadow prices in a perturbed system. In addition, the authors applied this concept to a recently published method termed Temporal Expression-based Analysis of Metabolism (TEAM), (Collins et al., 2012). This approach combines DFBA with GIMME in order to predict time-dependent flux profiles and extracellular metabolite levels. They were able to show that the shadow prices of the TEAM formulation hint at metabolites whose levels should rise or drop in order to increase consistency between flux predictions and gene expression data.

An advantage of the approach is that it allows for the simultaneous investigation of transcriptomics as well as metabolite data without the need to consider time-displacement between those data types (Nicholson et al., 2002, 2004). Since in the approach metabolite levels are characterized by their coefficient of variation over a given time-series the time delay between transcriptional regulation and its taking effect on the metabolic level does not need to be considered. However, the approach does not allow for the prediction of the levels of single metabolites. All predictions rather have to be considered as general trends.

### In planta—variability of metabolite levels is linked to differential metabolic pathways in *Arabidopsis's* responses to abiotic stresses

A recent study links predictions from the analysis of time-series transcriptomics data to metabolite levels (Töpfer et al., 2014). Similar to the Flux imbalance analysis described above, metabolite data are not directly integrated to make predictions, but are rather used to infer underlying organizational principles. This study relies on the findings of the integration of time-resolved transcriptomics data capturing the response of *A. thaliana* to eight different environmental cues. The predictions pertain to pathways which are differentially regulated with respect to a data-driven null model (Töpfer and Nikoloski, 2013; Töpfer et al., 2013). The study demonstrates that substrates of those differential metabolic pathways show on average a lower temporal fluctuations than other groups of metabolites. Moreover, these pathways include on average fewer substrates which are better connected than the rest of the metabolites. These observations not only underline the predictive power of transcriptomics data to make inferences on the level of the metabolites, but also relate results from constraint-based optimization approaches to topological network properties.

## Flux-sum to analyze metabolite turnover

### The flux-sum approach

The Flux-sum approach was developed with the idea of incorporating and investigating the metabolic state of a metabolic network model rather than only to focus on the flux distribution(s) (Chung and Lee, 2009). The flux-sum is a descriptor of a turnover rate of a metabolite and is given by summing up the incoming and outgoing fluxes of the reactions in which the metabolite participates as a product or a substrate, respectively. The algorithm involves calculating a basal flux-sum for each metabolite based on a flux distribution which maximizes an assumed objective, determining the maximum flux-sum of individual metabolites irrespective of an objective, and using the calculated bounds to manipulate the behavior of flux-sums of individual metabolites and to investigate their influence on the objective function. The approach was used to investigate different types of metabolite essentiality in *E. coli* and it was demonstrated to complement the reaction-centric view.

#### In planta—application of flux-sum to analyze nitrogen metabolism in a maize leaf model

A second-generation model of maize leaf has been recently investigated with the help of the flux-sum approach (Simons et al., 2014). To this end, the directional changes of the flux-sum of individual metabolites between two different nitrogen conditions in a wild-type maize leaf were qualitatively compared to the directional changes in the experimentally measured concentration levels. Therefore, this study used the flux-sum as a proxy for the metabolic pool size rather than its turnover. Since the flux-sum can vary in alternative optima, the authors only considered those metabolites whose ranges for flux-sums (normalized by the biomass rate) did not overlap between the compared scenarios. The study shows that inclusion of transcriptomics and proteomics data may result in flux-sums that better match the changes in metabolite pool sizes than without data integration.

## Future challenges and perspectives

This systematic review of constraint-based approaches for integrating metabolite data demonstrates a great potential of considering metabolite profiles at different levels of metabolic networks of varying size, ranging from small-scale to genome-scale reconstructions, and different level of details regarding the functional form of fluxes. The brief description of the approaches also indicates that the challenges of integrating metabolite data into metabolic networks depend on the coverage and spatio-temporal resolution of metabolite profiles, the quality of the network models used, as well as the particularities of the employed optimization approaches. In this section, we focus on these three crucial points followed by a succinct perspective for future development of computational approaches that rely on metabolomics data to investigate the behavior of biological systems.

### Subcellular compartmentalization and metabolic profiles

In contrast to prokaryotes, eukaryotic cells contain membrane-enclosed subcellular compartments, *e.g*., mitochondria, endoplasmic reticulum, and chloroplast. Therefore, the interpretation of currently available metabolite data from eukaryotic cells is complex. Currently, compartmentalization is only considered in a few studies (Masakapalli et al., 2010; Oikawa et al., 2011; Nägele and Weckwerth, 2013) and metabolite profiles are usually obtained on a tissue or organism level. In plant science, non-aqueous fractionation has been used to separate organelles (*i.e*., cytosol, plastid and vacuole) in a continuous non-aqueous fractionation density gradient prior to detection (Gerhardt and Heldt, 1984). The approach depends strongly on a bioinformatics evaluation to obtain reliable results (Klie et al., 2011).

The availability of the metabolic composition of each compartment will lead to a better understanding of pathways, their partitioning between compartments, and the multitude of necessary intracellular transport processes. This will contribute to metabolic networks of increasing quality and thus, to more accurate flux predictions.

Detection of a metabolite in compartments where the metabolite cannot be synthesized will help elucidating the existence of possible metabolite transporters between subcellular compartments (Lunn, 2007; Sweetlove and Fernie, 2013). Although new metabolite transporters are steadily identified (Sweetlove and Fernie, 2013), they have been so far only barely discussed in the context of constraint-based modeling (Mintz-Oron et al., 2009). As a result, for many metabolite transporters included in metabolic network reconstructions no evidence is available, but they are included to provide an operating network (Thiele and Palsson, 2010).

Finally, the metabolome is not a static feature, but changes during the life history of an organism. Therefore, careful spatio-temporal characterization of the metabolome can have a tremendous effect on the understanding of the temporal (in) activation of reactions in response to external and internal cues (Kim and Reed, 2012; Töpfer et al., 2012).

### Metabolic networks

The applicability of stoichiometry-based modeling approaches is highly dependent on the scale and quality of the analyzed metabolic network. Common issues encountered during the network reconstruction include: dead-end metabolites, blocked reactions, unknown co-factor specificity, and unknown reaction directionality. Dead-end metabolites are metabolites which are either only produced or only consumed in a metabolic network. During the refinement process of a metabolic reconstruction, the number of occurring dead-end metabolites is reduced by gap-filling algorithms (Satish Kumar et al., 2007). These algorithms add reactions from external databases (*e.g.*, KEGG Kanehisa et al., 2012), allow reactions to operate in their reverse direction, or add unverified transport reactions to the network. Therefore, these additions/modifications may often not be supported by the underlying annotation of the genome and can reduce the accuracy of the obtained predictions. Up to a certain network-scale, the model-discrimination approach of Kleessen et al. (2012) can facilitate validation for additional and modified reactions by finding the best support based on measured data. In addition, dead-end metabolites are associated to blocked reactions, which cannot carry any flux under the steady-state assumption.

The lack of knowledge about the biological system which is reconstructed also leads to inaccuracies of single reactions. For instance, for the majority of enzymes the specificity of co-factor usage (*e.g.*, NADP or NAD) is unknown. In addition, the lack of biochemical data can result in uncertainties in assigning correct (condition-specific) directionality to a reaction which can have a significant impact on the network's performance (Haraldsdóttir et al., 2012). Finally, the structure of biochemical pathways is well-established only for the central metabolism of model organisms (Breitling et al., 2008). Thus, the modeling results for less-characterized organisms and for pathways not included in central metabolism have to be treated with care, especially it the predictions are not driven by data integration.

### Alternative optimal solutions

Optimization problems, such as those typically encountered in constraint-based modeling, can result in non-unique flux distributions for a unique optimum value of the assumed objective. Therefore, to understand the quality and robustness of predictions, the alternative optima need to be investigated. In general, for constraint-based modeling approaches, two ways can be applied to deal with alternative optima (Mahadevan and Schilling, 2003; Sweetlove and Ratcliffe, 2011): (i) analyze flux ranges (*e.g.*, with help of flux variability analysis (FVA) Burgard et al., 2001) or, if possible, to enumerate all alternative solutions; (ii) consider additional objectives to obtain a unique solution. To find all alternative optima for a constraint-based modeling approach usually the introduction of (additional) integer variables is required (Lee et al., 2000). Therefore, this approach often results in computational problems which are not tractable for genome-scale networks. In contrast, a unique solution or, at least, a narrowed down solution space can be obtained by a two-step objective. In this approach, the general optimization problem, *e.g.*, FBA, is solved in the first step. A second objective (*e.g.*, minimization of the sum of fluxes Holzhütter, 2004; Lewis et al., 2010) is then optimized for the solutions achieving the optimum value of the first step. Finally, the integration of additional constraints into the optimization problem is expected to further reduce the space of solutions (Reed, 2012).

### Future directions

With the ever-increasing quality of data from high-throughput technologies, these data can be readily employed to obtain more accurate metabolic network reconstructions. One promising future direction is to consider more refined methods to analyze condition-specific thermodynamic properties which may have a large effect on the resulting predictions.

In addition, plants experience a natural day-night cycle which implications are rarely investigated in the context of large-scale modeling (Cheung et al., 2014). Therefore, to analyze the behavior of plant systems in a specified condition, one needs to design a multiscale model, including various cell types, their interactions, and responses with respect to naturally occurring cycles and conditions (Arnold and Nikoloski, 2014).

Addressing these perspectives necessitates the development of additional refined approaches which can bridge the gap between statistical approaches applied in data analysis and the mechanistic large-scale view taken in the constraint-based modeling framework. Furthermore, in the future a transition from the usage of absolute metabolite levels, favored and necessary in kinetic modeling, to the easier to obtain relative levels usually reported in metabolomics studies in plants and animals has to be considered in research efforts.

Finally, constraint-based modeling approaches are currently hampered by the validation of the predicted flux distributions. Fluxes can only be estimated by using various isotope labeling approaches (Schuetz et al., 2007; Williams et al., 2010) which are currently not applicable on a genome-scale level despite the developments of genome-scale carbon maps (Ravikirthi et al., 2011). New technologies and computational methods will be necessary to facilitate the evaluation of the flux predictions and to determine the actual degree to which various data types (from transcriptomics, proteomics, and metabolomics technologies), their combination and positon in the network can help to constrain the flux distributions and to delineate their relationship to metabolomics data profiles.

### Conflict of interest statement

The authors declare that the research was conducted in the absence of any commercial or financial relationships that could be construed as a potential conflict of interest.
